# Recent advances in lymphatic targeted drug delivery system for tumor metastasis

**DOI:** 10.7497/j.issn.2095-3941.2014.04.003

**Published:** 2014-12

**Authors:** Xiao-Yu Zhang, Wei-Yue Lu

**Affiliations:** ^1^Key Laboratory of Smart Drug Delivery (Fudan University), Ministry of Education, School of Pharmacy, Fudan University, Shanghai 201203, China; ^2^Key Laboratory of Molecular Engineering of Polymers, Department of Macromolecular Science, Fudan University, Shanghai 200433, China

**Keywords:** Lymphatic metastatic tumor, lymphatic system, metastasis, targeted drug delivery system, liposome, nanoparticle, polymer micelle (PM)

## Abstract

The lymphatic system has an important defensive role in the human body. The metastasis of most tumors initially spreads through the surrounding lymphatic tissue and eventually forms lymphatic metastatic tumors; the tumor cells may even transfer to other organs to form other types of tumors. Clinically, lymphatic metastatic tumors develop rapidly. Given the limitations of surgical resection and the low effectiveness of radiotherapy and chemotherapy, the treatment of lymphatic metastatic tumors remains a great challenge. Lymph node metastasis may lead to the further spread of tumors and may be predictive of the endpoint event. Under these circumstances, novel and effective lymphatic targeted drug delivery systems have been explored to improve the specificity of anticancer drugs to tumor cells in lymph nodes. In this review, we summarize the principles of lymphatic targeted drug delivery and discuss recent advances in the development of lymphatic targeted carriers.

## Introduction

As a relatively new branch of targeted therapies, lymphatic targeted drug delivery systems are currently eliciting much attention. These systems are used to improve the curative therapeutic effect of chemotherapy drugs and are applied to enhance the mucosal immunity and oral absorption of macromolecular drugs.

According to the Globocan 2012, 65,950 people were diagnosed with Hodgkin’s lymphoma worldwide in 2012, and 25,469 people died of the disease. In the same year, 385,741 people were diagnosed with non-Hodgkin’s lymphoma worldwide and 199,630 people died from the disease. The lymphatic system is the first site of metastasis for most solid tumors, and lesions indicate pathological changes associated with various diseases. However, the unique anatomical properties of the lymphatic system generally make it difficult for medicines to reach the targeted site in the lymphatic tissues, including lymph node metastases^1^. Therefore, an effective lymphatic targeted drug delivery system is urgently needed to deliver drugs to the lesions successfully with improved therapeutic efficacy^2^^,^^3^.

Recent studies have revealed the potential of drug-loaded liposomes, nanoparticles, and micelles, which can deliver a drug into lymphatic tissues, including lymph node metastases, and then release the drug inside these lesions. Consequently, the drug could effectively treat the disease, and its side effects to healthy organs could be reduced as well^4^. In this review, we used representative examples to illustrate the potential of a lymphatic targeted drug delivery system for tumor metastasis.

## Characteristics of the anatomy and physiology of the lymphatic system

At the beginning of the 17^th^ century, the lymphatic system was first described by Gasparo Aselli, who found “milky veins” in the mesentery of “well-fed” dogs. The lymphatic system is a complex network that consists of the lymphatic vessels, lymph nodes, spleen, thymus, Peyer’s patches, and tonsils; this system has an important role in immune surveillance and response^5^. Meanwhile, the lymphatic system functions interdependently with the cardiovascular system and serves as the secondary vascular system in the body of vertebrates^6^^,^^7^. Unlike the cardiovascular system, the lymphatic system is a one-way, open-ended transit network without any central driving force^7^. The system begins in the dermis with the initial lymphatic vessels, which are blind-end lymphatic capillaries that are roughly equivalent in size to blood capillaries but much fewer in number^8^. The lymphatic capillaries consist of a single layer of thin-walled, non-fenestrated lymphatic endothelial cells (LECs). LECs have poorly developed basement membranes, which sometimes poorly perform and lack tight and adherens junctions^5^. Lymphatic capillaries are extremely porous because the gaps between LECs are roughly 30-120 nm in size, allowing the entry of large particles, cells, and interstitial fluid^9^. Meanwhile, the gaps between blood capillaries are less than 10 nm. Several hydrophilic channels are present in the tissue space (approximately 100 nm). Given the physiological characteristics of the tissue space, drug delivery systems (<100 nm) can be guided into the lymphatic system via an injection through the tissue space. In the lesions caused by inflammation or cancer, the gap among LECs would be larger (300-500 nm) and would allow particles of appropriate sizes to enter the lymphatic system^8^^,^^10^.

Other investigations have found that the lymphatic system has a crucial role in the recognition and response of the immune system to disease; most solid cancers initially spread from the primary site via the tumor’s surrounding lymphatic tissue before hematological dissemination^11^.

Generally, orally or intravenously administered drugs first enter the blood circulation before they are distributed throughout the body, including the related lesions. Inevitably, this traditional administration route causes drugs to rapidly accumulate in normal organs and tissues, which may cause side effects. Given this dilemma, lymphatic administration possesses obvious advantages. Drugs administered via the lymphatic system could be more easily distributed in the lymphatic system and less in blood circulation, which helps strengthen the therapeutic effects and reduce the systemic toxicity^12^. This approach is particularly useful for lymphatic diseases, such as inflammation and tumor metastasis, where intravenously administered drugs rarely enter the lymphatic system.

## Mechanism of lymphatic targeted drug delivery system

**Figure 1:**
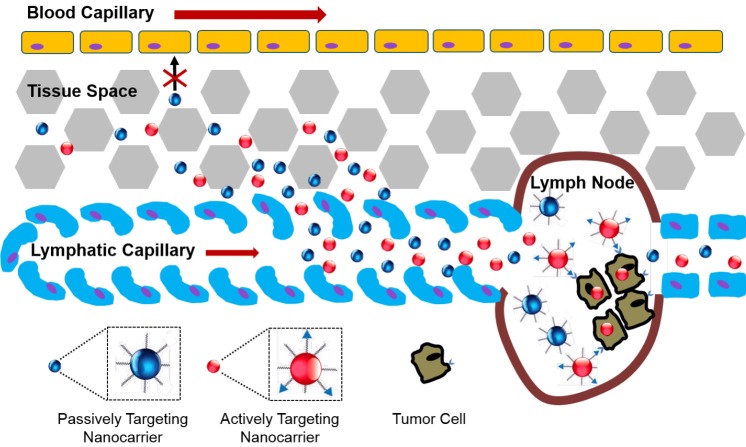
Lymphatic targeted mechanism of nanocarriers. Nanocarriers in tissue space are guided to the lymphatic capillary and accumulate in the lymph nodes (Reprinted with permission from Kerjaschki D^13^).

Lymphatic targeted therapy for tumor metastasis can be divided into passive and active targeting therapy^13^ (**Figure 1**). Different nano-sized drug delivery systems, such as solid lipid nanoparticles and nanocapsules, can reach the target position via a passive targeting mechanism. Active targeting can be realized by biochemical interactions, such as the affinity between the receptors on tumor cells and their ligands or between the antigens on tumor cells and their respective antibodies.

### Passive lymphatic targeting

Passive targeting refers to the use of the structural features of the lymphatic system for drug transport. For example, orally administrated nanoparticles and chyle particles will be absorbed by Pye’s lymph nodes, whereas subcutaneously injected ones will be absorbed by the lymphatic and lymph nodes. Particles with different sizes can be targeted to different parts of the body. The intravenous administration of emulsion droplets (with sizes ranging from 0.1 to 0.5 μm) is vulnerable to clearance by the macrophages in the reticuloendothelial system (RES) of the liver, spleen, lung, and bone marrow^14^. Fat emulsion droplets do not enter liver circulation. Most of these droplets optionally aggregate within the macrophages in the RES, thereby realizing effective drug targeting to the lymphatic system to improve the anti-inflammatory effects. The earliest drug that was used for lymphatic targeting was Mitomycin C, which can be delivered via intraperitoneal or subcutaneous injection in the form of water in oil (W/O) or oil in water (O/W) emulsions. Previous studies have shown that the lymphatic targeting of W/O emulsions after local injection is better than that of O/W emulsions. If a drug is loaded into nanoparticles before it is dispersed into oil emulsions, its lymphatic targeting can be significantly enhanced^15^^,^^16^.

When monolayer endothelial cells form capillary lymphatic vessels in the process of lymph formation during cancer or inflammation, the basement membranes of lymphatic vessels are incomplete and highly permeable. Moreover, lymphatic drainage channels (normally 30-120 nm) may be further expanded to 300-500 nm^8^^,^^10^. By contrast, the gaps between blood capillaries are less than 10 nm. In addition to transport via the bloodstream, macromolecules and colloids of suitable sizes (<100 nm) will also be brought into the lymphatic drainage by diffusion through these lymphatic drainage channels. However, these macromolecules or colloids may be captured by macrophages when they flow through the lymph nodes. Therefore, the passive targeting of lymph node metastases can be achieved in this manner.

### Active lymphatic targeting

Active targeting is mostly based on ligand-mediated endocytosis. In the process of tumor development, various vascular endothelial growth factors (VEGFs) are secreted by cancer cells; these VEGFs can bind with corresponding receptors on the LECs and induce the growth of tumor lymphatic vessels, thereby promoting tumor metastasis via the lymphatic system^17^. He *et al*.^18^ chemically synthesized calcium carbonate (CaCO_3_) nanoparticles with an average diameter of 58 nm and an average zeta-potential of +28.6 mV. This nanoparticle carried small interfering RNAs (siRNAs) against VEGF-C. Compared with the calcium carbonate nanoparticles containing nonspecific siRNA, nanoparticles with siRNA-targeting VEGF-C showed higher transfection efficiency in the human gastric cancer cell line SGC-7901, which was demonstrated by an ~80% decrease in the concentration of VEGF mRNA and VEGF-C. Animal experimental results showed that the CaCO_3_ nanoparticle-siRNA-VEGF-C complexes could inhibit lymph angiogenesis, regional lymph node metastasis, and growth of the primary tumor. The tumor lymphatic metastasis rate was 70% in the control group, whereas that in the test group was only approximately 20%.

Recently, researchers have found a cyclic peptide, LyP-1^19^, with nine amino acids. This peptide can specifically bind to tumor cells, tumor lymphatic cells, and tumor-associated microphages. Yan *et al*.^20^ constructed a LyP-1-conjugated liposome drug delivery system for targeting lymphatic metastatic tumors. These liposomes are drained into the lymphatic system after subcutaneous or intramuscular injection. LyP-1-mediated targeting effect could increase liposome uptake by tumor metastatic lymph nodes. Antitumor drugs released from these liposomes could kill the cancer cells and damage the lymph vessels in lymph nodes and tumor-associated macrophages. Consequently, this LyP-1-conjugated liposome drug delivery system obviously enhanced the inhibition effect of lymph node metastasis.

## Carriers for lymphatic targeted therapy

The penetration of particles is generally inversely proportional to the size. Smaller particles (<100 nm) find it easier to penetrate the microvasculature of lymphatic metastatic tumors, thereby enhancing the anti-tumor efficiency. Furthermore, drugs can be passively targeted to the lymphoma through the high accumulation of nanometer carriers during lymphatic metastasis. Simultaneously, nanocarrier surfaces can be modified for active targeting^21^.

Ideal lymphatic targeted nanometer drug delivery systems should meet the following criteria: high uptake by the lymphatic system, release and accumulation of drug during metastasis, and low toxicity to normal tissues. Nano drug delivery carriers that target the lymphatic system in recent literature include liposomes, nanoparticles, macromolecule polymers, polymer micelles (PMs), activated carbons, silicon, and nano-emulsions, among others. In this review, two types of carriers are discussed in detail.

### Liposomes

A liposome is a type of drug delivery system composed of a lipid bilayer. These structures can be loaded with drugs by entrapping hydrophilic drugs into the aqueous core or entrapping the lipophilic drugs between the phospholipid bilayer. Given its affinity to biological membranes, liposomes naturally have lymphatic targeting properties, as shown by their selected targeting to lymphoid tissue through intraperitoneal, subcutaneous, and intramuscular injections. After coming into contact with tumor cells or being engulfed by these cells, a liposome can release the drug it carries and show a long-term effect. Lymphatic uptake is related to the particle size, composition, and lipid dose of liposome. Based on their natural features, easy modification, well-controlled size, and feasible drug loading, liposomes have been regarded as the most suitable type of nanometer carriers.

Liposomes have shown unique advantages in lymphatic targeting treatment in recent studies. By manipulating formulation lipids with different fatty acid chain lengths, temperature- or pH-sensitive liposomes can be constructed to permit the controlled release of their contents^22^. Ling *et al*.^23^ evaluated oral delivery of a poor bioavailable hydrophilic drug called cefotaxime in three different forms: liposomal formulation, aqueous free drug, and a physical mixture of the drug and empty liposomes. They showed that the liposomal formulation leads to a significant enhancement of the lymphatic localization of the drug relative to the other two formulations. Latimer *et al*.^24^ developed liposomes of paclitaxel and a vitamin E analog, a-tocopheroyloxy acetic acid (a-TEA), in an aerosol formulation for treating murine mammary tumors and metastasis. Moreover, hyaluronic acid (HA) has been widely investigated for its unique properties. HA is a negatively charged hydrophilic polymer of linear glycosaminoglycan composed of glucuronic acid (GlcUA) and N-acetyl-D-glucosamine (GlcNAc) molecules that are linked by alternating β-1,4 and β-1,3 glycosidic bonds^25^. Ye *et al*.^26^ prepared high-molecular weight hyaluronic acid-modified docetaxel-loaded liposomes (HALPs) and low-molecular weight hyaluronic acid-modified docetaxel-loaded liposomes (LMWHA-LPs) via electrostatic attraction. A series of *in vivo* and *in vitro* experiments showed that LMWHA-LPs could be employed as potential lymphatic targeting carriers for diagnosis and treatment.

Further research has revealed that in the process of lymphatic targeting drug delivery, liposomes cannot be completely absorbed by the lymphatic system; a portion of these liposomes is situated in the interstitial injection site^27^. Moghimi *et al*.^28^ proposed that the interaction between the surface of some liposomes and interstitial proteins may increase the size of liposome, such that liposomes could not be completely absorbed by the lymphatic system; instead, they can be stranded at the injection site. Compared with the non-modified liposomes, the spatially stabilized PEGylated liposomes also showed no obvious improvement in lymphatic absorption. This phenomenon may be explained by the existence of a PEG hydrophilic layer. PEGylation of liposomes would hamper the phagocytosis by macrophages and influence their uptake by the lymph nodes. Therefore, in addition to the space stability of liposomes and macrophage phagocytosis, other factors may affect the lymphatic absorption and uptake of liposomes.

The accumulation of liposomes is caused by mechanical retention or macrophages of liposomes in normal lymph nodes; this process may damage normal lymphatic tissue^29^. Previous researchers used polyoxyethylene chains or ethoxycarbonyl copolymer support clips^30^ to modify the surface of liposomes, thereby enabling the liposomes to evade phagocytosis by macrophages in the lymph nodes^31^. Feng *et al*.^32^ combined “pressure compensation” and “avoidance of macrophage ingestion” strategies by mixing PEGylated liposomes with a cationic polysaccharide (DEAE-dextran). Both the cationic polysaccharide and the negatively charged tissue clearance under physiological conditions produced the required osmotic pressure to speed up the drainage of liposomes into the lymphatic system, reduce liposomal local retention at the administration site, and lower liposomal uptake by normal lymph nodes. These strategies effectively prevented possible damage to normal tissues and increased the amount of liposomes in the blood.

In general, the accumulation of liposomes in lymph nodes is a passive process. Liposomes alone cannot discriminate between normal and metastatic lymph nodes. The uptake of liposomes by metastatic lymph nodes is lower than that by normal lymph nodes because of the structural damage in metastatic lymph nodes. This problem has been addressed in recent reports by modifying the drug delivery systems with the cyclic peptide LyP-1^33^^-^^35^, which contains nine amino acids; this peptide can specifically bind with tumor cells and the p32 receptors that are highly expressed on the surface of tumor lymphatic vessels. The LyP-1-conjugated liposome drug delivery system constructed by Yan *et al*.^20^ showed good targeting ability to lymphatic metastatic tumors after subcutaneous injection. The specific binding of the LyP-1, PEG, liposome with tumor metastases, tumor lymphatics, and tumor-associated macrophages enhanced the inhibitory effect on lymphatic metastases and improved the suppressing effect on lymphatic metastasis. All these data indicated that the LyP-1-conjugated liposome drug delivery system may provide a promising actively targeting drug delivery platform for the treatment of lymphatic metastatic tumors.

### Nanoparticles

Nanoparticles, nanospheres, and nanocapsules are all solid colloid particles made from natural or synthetic polymer materials; these structures have an average particle size of 10-1,000 nm. Nanocapsules are made of polymeric shells with a liquid core, wherein the drug is dissolved. Nanoparticles and nanospheres are both matrix carriers, which are solid spheres or particles, such that the drug is absorbed on its surface, wrapped around it, or dissolved in its core. Currently, nanospheres and nanocapsules are collectively referred to as nanoparticles. Nanoparticles have several advantages, such as their slow drug release, long circulation, good stability, safety, and high efficiency.

Most nanoparticles are made from natural or synthetic biodegradable polymers. Natural materials include proteins, gelatin, peach gum, chitosan, and HA. Synthetic materials mainly include polyalkyl cyanoacrylate (PBCA), polylactic acid (PLA), poly (lactic-co-glycolic acid) (PLGA), and polymethyl methacrylate (PMMA). To improve the lymphatic targeting efficiency of the anti-cancer agent vincristine sulfate (VCR), Tan *et al*.^36^ prepared poly (butyl cyanoacrylate) nanoparticles (VCR-PBCA-NPs) by emulsion polymerization and superficially modified these VCR-PBCA-NPs with Pluronic F127. Significant (*P*<0.05) and very significant (*P*<0.01) improvement in the lymphatic targeting efficiency of VCR was achieved by VCR-PBCA-NPs and F127-VCR-PBCA-NPs, respectively, compared with the VCR solution.

Active drug targeted delivery systems modified by small molecules like nanoparticles on their surface can increase the binding specificity, thereby ensuring that the specified drug carriers reach the target sites^37^. Therefore, active targeting systems combined with nanoparticles can be promising tools for drug delivery. Fan *et al*.^37^ conjugated the polypeptide of the follicle-stimulating hormone (FSH) to a nanoparticle (FSHP-NP) to target FDH receptors (FSHR) in lymphatic metastases of ovarian cancer. A paclitaxel (PTX)-loaded FSHP-NP (FSHP-NP-PTX) was further developed, and its anti-tumor effect was determined *in vivo* and *in vitro*. Their results showed that FSHR is a novel therapeutic target for ovarian cancer and the delivery of PTX via FSHP-NP could represent a novel therapeutic approach to ovarian cancer.

HA is a typical naturally-originating biodegradable material for lymphatic targeted drug delivery. For example, breast cancer cells tend to take in large amounts of HA for the over-expression of P-glycoprotein during their growth. Cai *et al*.^38^ prepared pH-sensitive hyaluronic acid doxorubicin (HA-DOX) complexes, in which DOX was connected to HA by cleavable hydrazone bond. After peritumoral subcutaneous injection in tumor model animals, the HA-DOX complexes were transported from the orthotropic tumor to the lymphatic metastasis, resulting in improved anti-tumor activity, as shown by the 70% decrease in the tumor volume at 10 weeks after inoculation compared with the free DOX treatment. Compared with the death of all tumor-bearing nude mice in the free DOX group at 18 weeks after inoculation, at least 50% of the nude mice in the HA-DOX group survived in the entire observation period (24 weeks). Furthermore, no tissue damage or heart toxicity was observed in the HA-DOX group.

Active targeting modification is another current research focus together with the search for novel receptors and ligands related to the lymphatic metastasis. During tumor development, a variety of VEGFs are secreted by cancer cells; these ligands bind with corresponding receptors on the LECs, inducing the growth of tumors in lymphatic vessels and promoting tumor metastasis through the lymphatic system. In addition to the successful work of He *et al*.^18^, Luo *et al*.^39^ prepared LyP-1--modified PEG-PLA nanoparticles, which can specifically target lymphatic metastases. The distribution of LyP-1-modified nanoparticles in metastatic lymph nodes was eight times higher than that of non-modified nanoparticles after subcutaneous injection at the foot pad in the animal model bearing lymphatic metastases of pancreatic cancer^32^^,^^40^.

### Polymer micelles (PMs)

PMs have been developed since the 1990s and used as drug delivery carriers. PMs spontaneously form into thermodynamically stable systems with core-shell structures in aqueous solution. The inner core formed by PM hydrophobic fragments can load certain insoluble drugs, thereby improving the solubility of these drugs. The hydrophilic shell can protect drugs from outside adsorption or degradation and avoid phagocytosis by RES, such that PMs can prolong drug circulation time *in vivo*. The diameter of polymeric micelles resembles that of natural viruses; their sizes can vary from 10 to 100 nm^41^^-^^43^, which reduces their accumulation in the RES organs and aids in overcoming physiological barriers, such as the lymphatic transport to lymph nodes after intradermal injection^43^^,^^44^ extravasation, and their deep penetration and high accumulation in solid tumors after systemic injection^43^^,^^45^. Furthermore, PMs can realize passive targeted drug delivery, but they can also be connected to specific ligands for active targeted drug delivery. Therefore, PMs can significantly improve drug bioavailability and reduce their side effects.

Polyethylene glycol phospholipids (PEG-PE) have been widely studied in recent years because they are approved by the FDA, such that they can be used in the human body as drug delivery carriers. Wang *et al*.^46^ systematically prepared two PEG-PG micelles: doxorubicin-loaded micelles (M-DOX) and vinorelbine-loaded micelles (M-VNR). Their *in vivo* antitumor activity was then studied. Compared with free drugs, the drug-loaded micelles can increase the amount of drugs that reach the cells by changing the membrane fluidity; these micelles can also increase the cellular uptake of drugs by adjusting the genes that control protein expression. In the mouse model for 4T1 breast cancer metastases, both M-DOX and M-VNR inhibited the tumors from lung metastases as well as reduced the systemic and acute toxicity of the free drugs by increasing drug distribution in the lymph nodes.

The small sizes of PMs (10-100 nm) make them suitable for lymphatic delivery, and several studies have examined their role in anti-metastasis therapy. Li *et al*.^47^ investigated the anti-metastatic effectiveness against malignant breast cancer by small-sized polymeric micelles (SPMs) with a mean diameter of 16.76 nm and loaded with docetaxel. SPMs were constructed of poly(D,L-lactide)_1300_-β-(polyethylene glycol-methoxy)_2000_ (mPEG_2000_-b-PDLLA_1300_). A 4T1 mouse breast cancer model was used to compare the therapeutic effectiveness of SPMs and free docetaxel (Duopafei) against breast cancer metastasis. Their results showed that SPMs could provide an enhanced method of docetaxel delivery in breast cancer metastasis, which may be a valid chemotherapeutic strategy for breast cancer patients with resected primary tumors.

## Conclusion

A lymphatic targeted drug delivery system with a unique physiological disposition provides an effective tool for the treatment of diseases related to the lymphatic system, such as tumor metastasis, inflammation, and infectious disease. Carriers of colloid particles are viewed as the main methods to realize lymphatic targeted drug delivery. The efficiency of lymphatic targeting may be improved by screening different carriers, formulations, and routes of administration. Taken together, the current lymphatic targeted drug delivery systems have achieved significant advances in various stages, but they still have some limitations. For example, their lymphatic uptake rate is not high and the biocompatibility of some carrier materials is not ideal for the lymphatic system, which may lead to some adverse reactions. Basic research on the mechanism of lymphatic targeting is underway. Several research proposals could be developed to study lymphatic targeted drug delivery systems. Future work can focus on the following aspects: (I) to make the drug delivery system actively target the lesions of interest, active targeting agents for the lymphatic system or metastatic lesions should be given more attention; (II) with respect to the carrier materials, novel materials need to be developed, especially biodegradable materials with active targeting function. In summary, we believe that a lymphatic targeted drug delivery system for tumor metastasis will be a promising approach to treat primary tumors of the lymphatic system and all types of lymphatic metastases.
